# Optimization extraction of rosemary essential oils using hydrodistillation with extraction kinetics analysis

**DOI:** 10.1002/fsn3.2549

**Published:** 2021-09-16

**Authors:** Hanyue Chen, Zhanying Gu, Ling Yang, Ruonan Yang, Yaxin Ji, Qingyang Zeng, Fangmeng Xiao, Peng Huang

**Affiliations:** ^1^ Central South University of Forestry and Technology Changsha China

**Keywords:** essential oil, extraction kinetics, first‐order kinetic model, hydrodistillation, rosemary

## Abstract

Rosemary (*Rosmarinus officinales* L. *(Labiatae)*) is one of the major economic crops in the world, and rosemary essential oil (REO) is one of the top products derived from rosemary and has excellent commercial prospects. Many factors affect the yield of REO extracted by hydrodistillation (HY). This study was proposed to identify and analyze these factors to maximize the yield of essential oils and reduce the cost. First, two different single‐factor extraction experiments were conducted, (1) adding NaCl and (2) using various organs of the plant, to determine the influence of each factor on the oil yield. Based on single‐factor experiments, the orthogonal experiments (L_9_, 3^3^) were designed to determine the optimal conditions for the extraction of rosemary oil. Meanwhile, the kinetic extraction analysis of the test data was carried out. The results revealed that the highest oil yield was achieved when rosemary leaves were crushed to 2 cm, the ratio of water to the material was 1:3, and NaCl concentration was 5%. A simple first‐order kinetic model has also proved to be an acceptable general choice and allows to predict the output of extraction operations overtime accurately and robustly in practice. This study provides a reference scheme for using hydrodistillation to extract rosemary essential oil.

## Graphical abstract

Using hydro distillation, the best processing conditions of rosemary essential oil extraction were determined by single‐factor experiments and orthogonal experiments, and the theoretical yield value under the best processing conditions was obtained by the first‐order kinetic model.

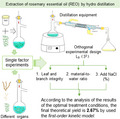

## 1 INTRODUCTION

Rosemary (*Rosmarinus officinalis* L.) is an essential perennial aromatic, evergreen shrub belonging to the family *Lamiaceae* and indigenous to the Mediterranean region (Naimi et al., 2017; de Oliveira et al., 2019). However, it could be found worldwide and grown widely in northern central Europe, America, and East Asia (Ahmed et al., 2016; Tigrine‐Kordjani et al., 2012). This plant has a height of up to 2 m, with branches full of green leaves that exude a characteristic fragrance (de Oliveira et al., 2019). Its health benefits have been recognized since ancient times, and it is the raw material of many culinary traditions (Bellumori et al., 2015; Spadi et al., 2021). Moore et al. (2016) mentioned that Rosemary extract has antioxidant, anti‐inflammatory, antidiabetic, and anticancer properties.

Widespread use of rosemary is to extract the essential oil. Rosemary essential oil (REO) is a colorless or pale‐yellow volatile liquid extracted from the branches and leaves with a characteristic odor. It consists mainly of monoterpenes such as 1,8‐cineole, camphor, and α‐pinene (Rašković et al., 2014). These compounds in REO make REO can be used in many aspects. REO is considered a natural antioxidant, and it has been reported as an inhibitor of lipid oxidation in meat products (Sebranek et al., 2005). Furthermore, REO can be used for treating dyspepsia and mild spasmodic disorders of the gastrointestinal tract (Raskovic et al., 2015), and extensively used in industry, especially in the flavor and cosmetics industries, as additives in active packaging and in agriculture as additives to repel insects and sometimes acting as pheromones (Silvestre et al., 2019).

These characteristics, as well as consumers' increasing demand for healthy natural products, make REO a commercial product with wide application and high economic value (Spadi et al., 2021). REO accounts for about 1%–2.5% of the total plants. Like other essential oils, their quantity and quality are affected by various internal and external factors. In particular, the content of its chemical composition varies with the geographical region where plants grow, climate, plant parts used, and extraction methods (Borges et al., 2019). Current methods for extraction of rosemary essential oil include hydrodistillation, steam distillation, ultrasound‐assisted extraction (Heck et al., 2018), molecular distillation, adsorption, supercritical water extraction, CO_2_ supercritical extraction (Allawzi et al., 2019), and enzyme‐assisted extraction (Nadar et al., 2018). Hydrodistillation (HY) is the simplest method and commonly used to achieve industrial production. In HY, plant materials containing essential oils are placed in a distiller mixed with water (Spadi et al., 2021). A heat source heats the mixture, make them undergo physical alterations, and oil vapors along with water vapors come out due to thermal diffusion (Solanki et al., 2018), and the steam that is created passes through a condenser that allows the recovery of essential oil (Spadi et al., 2021). This procedure achieves component isolation according to their degree of hydro‐solubility rather than their boiling points (Presti et al., 2005). Since the cell wall provides the maximum resistance, this process takes long extraction, relatively high solvent consumption, and usually irreproducibility (Mohamad et al., 2019; Solanki et al., 2018).

Although this method has been used for a long time, few scientific research and operating conditions for REO extraction, some ways, mention the use of pressurized heating or the addition of organic solvents to promote extraction, but this does not meet the premise of saving energy and protecting the environment. Therefore, the extraction conditions are significant for maximizing the yield of REO and optimizing parameters such as energy, time, raw materials, and solvents (de AR Oliveira et al., 2016). Many factors affect the extraction process of HY. Adding NaCl to the water from which essential oil is extracted is an influencing factor, improving the REO yield during extraction. The oil content in organs of different parts of plants is different, so it is also a key factor. The solid/liquid ratio is another critical factor. Optimizing this ratio should maximize REO production and reduce solvent consumption, thus improving economic and environmental efficiency (Spadi et al., 2021). Finally, a study (Smallfield et al., 2001) reported that pretreatment, such as crushing raw materials, can improve the yield. Therefore, it is necessary to study the joint influence of these operating factors to maximize the recovery rate of REO.

In addition, distillation time (DT) is known as also very important (Spadi et al., 2021). Many studies have modeled the extraction process of volatile essential oils and simulated the kinetics by making many assumptions (Ait Amer Meziane et al., 2019). When dealing with the relationship between the yield of essential oil and distillation time by kinetic data, two types of curves were revealed: exponential curve and S curve, while HY belonged to the exponential curve (Benyoussef et al., 2005).

In this context, we aimed to optimize the method for extracting rosemary essential oil based on the HY method under different designed experiments. The DT is an independent variable to carry out the first‐order kinetic modeling. The main objectives of this study were (1) to investigate the effect of adding sodium chloride (NaCl) on the extraction of rosemary essential oil, (2) to examine the difference in the amount of essential oil extracted from rosemary branches and leaves, (3) according to the data of the previous two single‐factor experiments, further to determine the optimal extraction of essential oil using orthogonal experiments, and (4) Lastly, considering the distillation time, different parts of the rosemary, the ratio of water‐to‐raw material, and concentration of NaCl as influencing factors, the kinetic extraction model describing the extraction process was successfully constructed.

## 2 MATERIALS AND METHODS

### 2.1 Study site

Materials for this study were collected from the Production and Research Base of Spice Plant Cultivation and Utilization (113.08°E, 27.78°N) of Central South University of Forestry and Technology (CSUFT), Zhuzhou, Hunan Province, China. The site is plain, with an average elevation of 60 m. The local climate is a typical humid, subtropical monsoon climate, with four distinct seasons, average annual precipitation of 1400–1700 mm, and an average yearly temperature of 16–18°C. Soil type is mainly acidic red loam (Marburg, 1994).

### 2.2 Plant material collection

Rosemary was the 4‐year‐old rooted cuttings in the study site. The total sampling area is about 0.67 ha, and the whole rosemary was conducted every 4 m along the diagonal line of the field. The entire rosemary plants were divided into leaves, 1‐year branches, and perennial branches to accommodate different experimental designs. Plant materials were collected randomly in March 2019, placed in a cooler, and sent immediately to the laboratory. The plant materials were placed in a refrigerator at 4°C to ensure freshness.

### 2.3 Single‐factor experiments

The two single‐factor experiments are independent of each other and have no interaction. Experiment 1 (E1) was an exploratory experiment; it examined the differences in oil yield related to the amount of NaCl added. The experiment used a combination of fresh rosemary leaves and branches. The rosemary material was mixed with NaCl and distilled water in a distillation flask, and hydrodistillation was continued for 3 h to ensure no more essential oil was obtained. Rosemary contained volatile oil experiences thermal stress due to thermal energy supplied by the water during hydrodistillation. This phenomenon leads to oil diffusion, which is then carried by water vapor generated during the process toward the condenser (Solanki et al., 2018). For extracting the oil–water mixture distilled from each condition, the extraction process started at room temperature (25°C) (Akhbari et al., 2018). The use of a separating funnel for direct extraction is considered in terms of environmental protection and economy, and no organic solvent is needed to assist. The ratio of water to the material was 1:3. The following concentrations of NaCl were assessed: 0 (control), 0.5, 1.5, 3.0, 5.0, and 5.5 (unit: %).

Experiment 2 (E2) examined the variance in oil yield from the different parts of the rosemary. In this experiment, the ratio of water to the material was 1:1, both of which are 400 (g/ml). Because the size and essential oil content of different parts of rosemary are different, the volume of perennial branches is larger than that of 1‐year branches and fresh leaves. However, the maximum bearing capacity of the hydrodistillation device used in the laboratory is 800–1000ml, so the solid/liquid ratio of 1:1 can meet the needs of extracting different parts of rosemary. In addition, after the exploration of experiment 1, the total distillation time of E2 was determined to be 2 h, and the amount of essential oil was recorded every 30 min. The distilled essential oil was bottled individually and labeled with the extraction date and experiment number. All subsequent experiments are distilled for 2 h. Both experiments were repeated three times to examine the experimental error. All the experiments use 220 V voltage in the first 15 min of distillation and change it to 100 V after the temperature of the distillation flask stabilizes.

### 2.4 Multi‐factor experiment

The orthogonal L_9_ (3^3^) design was used to optimize the extraction conditions. This design assumes that there is no interaction between any two factors. In this experiment, variables were determined based on the results of two single‐factor experiments. Factor A crushed rosemary leaves to verify the influence of particle size on rosemary essential oil extraction (unbroken, 2‐cm pieces, and 1‐cm pieces, respectively). Furthermore, factors B and C verified the ratio of water‐to‐raw material (this factor was divided into 1:1; 1:2, and 1:3) and NaCl concentration (1.5%, 3.0%, and 5.0%) on the yield of essential oil extracted, respectively. Table 1 shows the experimental design for extracting essential oil from rosemary.

**TABLE 1:** Factors and levels for the orthogonal experiments

Factors	Level	Level	Level
	1	2	3
A. Leaf and branch integrity of the current year	Unbroken	2‐cm pieces	1‐cm pieces
B. Ratio of water‐to‐raw material	1:1	1:2	1:3
C. Concentration of NaCl	1.5%	3.0%	5.0%

The whole experiment was performed twice with 18 extractions. Distillation was performed for 2 h each time, and the amount of essential oil was recorded every 30 min to facilitate later calculation. SPSS20.0 obtained the orthogonal table.

### 2.5 Kinetic model

This study is based on the first‐order kinetic model mentioned in Zhang et al. (2020):

(1) $$\frac{{\mathrm{dY}}_{t}}{d_{t}}=K_{1\;}\left(Y_{e}‐Y_{t}\right)$$

where *Y_e_* (%) and *Y_t_* (%) represent the yield of EO at equilibrium or at any time, respectively, *t* (min) represents the distillation time, *K*_1_ represents the rate constant.

According to the initial and boundary conditions: *Y_t_* = 0 at *t* = 0 and *Y_t_* = *Y_t_* at *t* = *t*, Equation (1) is rearranged to a linear equation:

(2) $$\ln\left(Y_{e}‐Y_{t}\right)=\ln Y_{e}‐K_{1}t$$

A nonlinearized form can be obtained from Equation (2), as follows:

(3) $$Y_{t}=Y_{e}\left(1‐e^{‐K_{1}t}\right)$$

According to the actual data obtained in experiments 2.3 and 2.4, after processing and analyzing with the first‐order kinetic model, it is known that the result of experiment 2.3 accords with the Equation (2) of linear regression in the first‐order kinetic model. In contrast, the result of the orthogonal experiment is more suitable for fitting with the nonlinear regression of Equation (3).

Therefore, we made the scatter plot of the yield of REO and distillation time (DT) of different rosemary parts in experiment 2.3. With time *t* as abscissa and REO yield (%) as ordinate, fitted the yield increment of essential oil extracted from fresh leaves, annual branches, and perennial branches by linear regression, and obtained three fitting equations describing the extraction increment and time (Wang et al., 2015). Furthermore, He and Tian (2003), Li et al. (2014) proposed a simple method describing the extraction process of volatile oil (Laplace transform method). The process of extracting volatile oil from plant cells by hydrodistillation is usually as follows: (i) It diffuses from the inside of cells to the inner wall of cells, that is, the gas–solid interface and (ii) volatile oil enters the gas phase through the gas–solid interface. Because the solubility of volatile oil in water is minimal, the transmission process of a small number of volatile oil molecules in the liquid phase can be ignored; (iii) volatile oil molecules entering the gas phase are taken out of the system by continuously generated water vapor and transferred into containers for collection and preservation. Then, used the best extraction conditions obtained by orthogonal test, and the data of essential oil yield and DT were fitted by nonlinear regression. Equation (3) is the BoxLucas1 model in the exponential function, and the kinetic extraction equation under this treatment is obtained by exponential fitting with Origin.

### 2.6 Statistical analysis

Descriptive statistical analysis and one‐way analysis of variance were used to analyze the data for the two single‐factor experiments. For the orthogonal experiments, Univariate Analysis of Variance in the general linear model was used to find the relevant values such as the extreme value (R) and *F*‐value of each factor. Oil yield for all experiments was calculated using the following equation:

(4) $$R_{\mathrm{eo}(\%)}=\frac{M_{\mathrm{eo}}\left(g\right)}{M_{d}\left(g\right)}\times 100$$

Where R_eo_ = REO yield (% w/w), M_eo_ = REO mass extracted (g), and M_d_ = dry mass of rosemary leaves (g) (Spadi et al., 2021).

The figure and all tables were created using Excel 2016 and Origin 9.65.

## 3 RESULTS AND DISCUSSION

### 3.1 Effect of NaCl on REO extraction yield

E1 is an exploratory experiment completed under the conditions of a material‐to‐water ratio of 1:3 and distillation time of 3 h. It is confirmed that NaCl concentration directly influences the oil production rate (Table 2). When the concentration of NaCl reached 3%, the extraction yield reached a maximum of 0.61%. When the concentration of NaCl reached 5%, the extraction yield reached 0.60%. This trend does not hold for increased NaCl concentrations; at 5.5%, the yield was only 0.36%. The extraction yield without NaCl was the lowest, at only 0.02%. Overall, with an increase in NaCl concentration, the oil yield first increased remarkably, then decreased.

**TABLE 2:** Oil yield related to concentration of NaCl^a^

Concentration of NaCl (%)	Yield (%)
0	0.02 ± 0.003^e^
0.5	0.20 ± 0.003^d^
1.5	0.55 ± 0.028^b^
3.0	0.61 ± 0.020^a^
5.0	0.60 ± 0.010^a^
5.5	0.36 ± 0.040^c^

Note

Based on one‐way analysis of variance. Values represent means ± *SD*.

^b,c, d, e^ Yields are significantly different from^a^ and from each other.

^a^ Yields at 3.0% and 5.0% are not significantly different.

In extracting essential oil, adding NaCl is a method thought to improve the oil yield, mainly because the NaCl can effectively target the analyte on a molecular level and enter the rapid extraction stage to significantly improve its analysis signal (Bibi et al., 2016). The results show that a certain amount of NaCl can promote the yield of rosemary essential oil, but it will inhibit the yield of essential oil beyond that amount. One reason for this inhibition is that, with the increase in salinity, the water content of rosemary leaves decreases, while the content of phenols, alkaloids, and brass compounds increases (Móricz et al., 2016). These substances are usually the main components of rosemary essential oil, and the phenols have good antioxidant properties, which is one of the medicinal uses for rosemary essential oil (Moore et al., 2016). However, excessively high salinity will increase the oxidative stress of organic components in essential oil, thereby inhibiting their extraction (Móricz et al., 2016).

This study demonstrates that, with a water‐to‐raw material ratio of 1:3, the addition of NaCl influences the oil yield. The extraction yields under the conditions of 3% and 5% NaCl are very high, reaching 0.61% and 0.60%. When NaCl concentration is 1.5%, the extraction yield is 0.55%; according to the results obtained in E1, therefore, it is recommended to use a concentration of NaCl between 1.5% and 5.0% for distillation extraction, which can save costs and ensure a high extraction yield and applied to the subsequent orthogonal experiment of three factors and three levels.

### 3.2 Variation in oil yield from different parts of the rosemary

**FIGURE 1:**
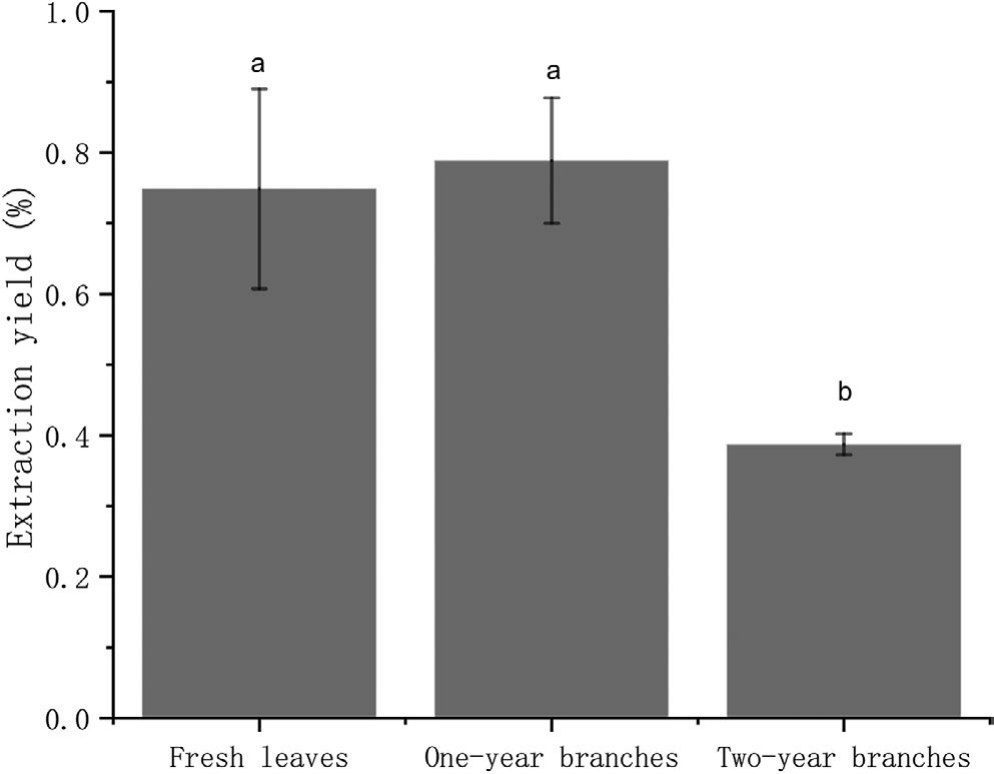
Oil yields from three different parts of the rosemary. *Error bars represent the standard deviation of the mean. ^a^Yields in fresh leaves and one‐year branches are not significantly different. ^b^Yields are significantly different from ^a^

The branches and leaves of rosemary contain aromatic oil (Rafael et al., 2011). Due to the essential oil glands located in the leaves and the flowers (Presti et al., 2005), rosemary essential oil is usually conducted by hydrodistillation of fresh leaves of rosemary annual flowering buds (Zheljazkov et al., 2015). Based on previous studies, REO is mainly located in leaves and flowers. It seems that few studies have mentioned whether rosemary branches can be used as the main raw materials for essential oil extraction. Therefore, in this study, we set up experiments to verify whether the 1‐year branches and perennial branches except leaves can extract essential oils. The oil yield of fresh leaves and 1‐year branches was sharply higher than perennial branches (Figure 1). In comparison, the average oil yield of 1‐year branches was slightly higher than that of fresh leaves. The mean difference is significant at the 0.05 level. The direct yield of 1‐year branches is slightly higher than that of fresh leaves, but the difference between the two factors is not significant (*p* > .05), so there is no comparative value. However, the results alone are also intriguing, proving that rosemary branches also contain many essential oils, especially the branches of the 1‐year. Our guess is that because rosemary has a unique plant morphology, many tiny leaves and unopened buds gather at the top of short branches to form a race. These microscopic parts containing essential oils are distilled together with the branches of the 1‐year, which makes the yield of branches of the 1‐year increase somewhat. This result can also inspire the follow‐up researchers and further explore the comparison between the 1‐year branches' yield results and fresh leaves.

However, as far as this experiment is concerned, from the point of view of saving cost and increasing yield, it can be advocated to extract essential oil from 1 year's leafy branches instead of just taking fresh leaves. In the subsequent orthogonal experiment, the 1‐year leafy branches of rosemary were used as experimental materials. The influence of crushing the one‐year leafy branches on the yield was further verified from pretreatment of experimental materials. In the long run, as a perennial economic plant, rosemary can survive for decades under suitable environmental conditions. Seasonal harvest of the rosemary leaves and branches is suggested to maximize product benefits.

### 3.3 Orthogonal experimental design generates optimum results

In E1 and E2, we found that adding NaCl to the solution can improve the yield in the process of hydrodistillation of rosemary, while the extraction yield of 1‐year branches and fresh leaves of rosemary is higher. Therefore, the rosemary raw material used in the orthogonal test is the leafy rosemary (including 1‐year branches and fresh leaves), and the influence of the pretreatment of crushing rosemary raw material on the yield is further discussed. Based on the single‐factor tests above, the concentration of NaCl and different parts of rosemary appear to be the significant factors that affect the yield of essential oil from rosemary. In the present study, these factors were examined using an orthogonal L_9_ (3^3^) test design. The test results are shown in Table 3, which indicates that the maximum yield of essential oil was 1.52%. However, we cannot select the best extraction conditions based only on the outcomes, and therefore, a further orthogonal analysis was warranted. Thus, the values of K, k, and R values were calculated and listed in Table 3. As seen from Table 3, the influence on the extraction yield of essential oil decreased in the order: B > C>A. The ratio of water‐to‐raw material was the most important determinant of yield according to the R values. The maximum yield of essential oil was obtained when the plant material crushed 2‐cm pieces, the water ratio to raw material was 1:3, and the NaCl concentration was at 5.0% (combination A2B3C3), respectively.

**TABLE 3:** Analysis of orthogonal experimental design

No.	A. Leaf and branch integrity of the current year	B. Ratio of water‐to‐raw material	C. Concentration of NaCl	Extraction yield (%)
1	1	1	1	0.67
2	1	2	2	0.47
3	1	3	3	1.52
4	2	1	2	0.89
5	2	2	3	0.74
6	2	3	1	1.05
7	3	1	3	0.62
8	3	2	1	0.57
9	3	3	2	0.92
K1	2.66	2.18	2.29	
K2	2.68	1.78	2.28	
K3	2.11	3.49	2.88	
k1	0.89	0.73	0.76	
k2	0.89	0.59	0.76	
k3	0.70	1.16	0.96	
R	0.19	0.57	0.20	

Note

K_1_ represents the average value of extraction yield from three experimental replicates under Level 1, and the same is true for K_2_ and K_3_ under Levels 2 and 3, respectively.

k_1_ represents the value obtained by dividing K_1_ by the test times under Level 1, and the same is true for k_2_ and k_3_ under Levels 2 and 3, respectively.

R_1_ refers to the results of the extreme analysis.

We next evaluated the *F*‐value and the significance level of each factor, as shown in Table 4 (*p* = .05). The results show that, although the water‐to‐material ratio has the most significant influence on the oil yield among all factors, the influence of the water‐to‐material ratio is not statistically different from the influence of the other factors.

**TABLE 4:** Test of significance of the orthogonal experiment

Factors	Deviation sum of squares	df	Mean Square	*F*	Sig.
A. Leaf and branch integrity	0.205	2	0.103	1.558	.391
B. Ratio of water‐to‐raw material	0.183	2	0.091	1.388	.419
C. Concentration of NaCl (%)	0.293	2	0.147	2.227	.310

Note

df, degrees of freedom; Sig., significant differences in the influence of factors on the extraction rate of essential oil. *p* = .05.

We examined different water‐to‐material ratios, blade integrity, and NaCl concentration in the orthogonal experiments of this study. The best processing conditions in the traditional sense are obtained by evaluating the range R, and the mean K. Borhan et al. (2013) mentioned the size of the particles that can interfere in the extraction process. The smaller the particle size, the higher the interaction between the plant sample and the solvent to obtain (de Oliveira et al., 2019). By comparing the k value of factor A, it can be seen that the extraction rate is the highest when the materials are crushed to 2 cm. Using the results of significance tests (Table 4), the *F*‐values of the three factors are more significant than Sig. (*p* > .05) indicates that factor A, leaf integrity, factor B, water‐to‐material ratio, and factor C, the NaCl concentration, have no significant effect on the oil yield.

In the comprehensive comparison, the water‐to‐rosemary ratio is the most significant factor affecting the oil yield, and the best level is 1:3. Therefore, in actual industrial production, we suggest considering both the addition of NaCl and the physical breakdown of the plant parts to achieve the optimal extraction conditions and the maximum oil yield.

### 3.4 Extraction kinetics

**FIGURE 2:**
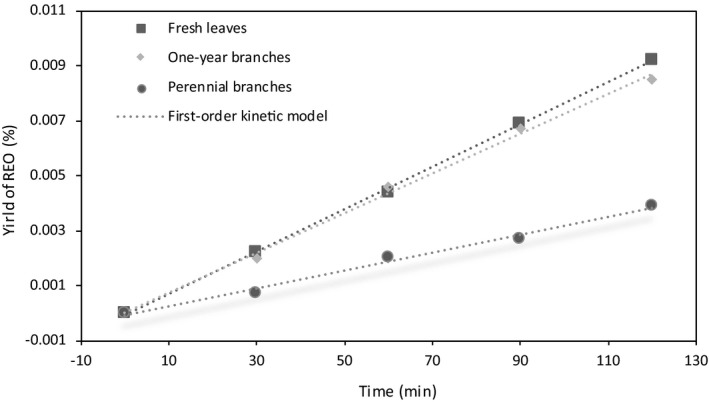
Kinetic curve for the yield of essential oil obtained by distilling different parts of rosemary. Points represent actual experimental data, and lines represent fitting behaviors predicted by first‐order kinetic model

**FIGURE 3:**
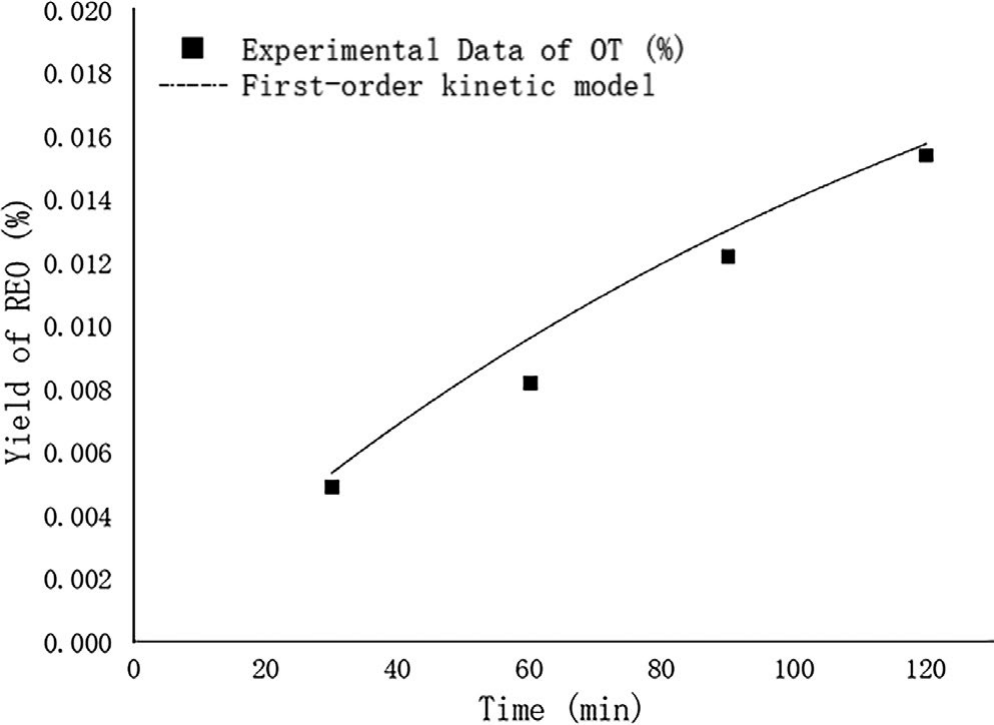
The first‐order kinetic curve of essential oil yield was obtained under A2B3C3 treatment. Points represent data, and lines represent fitting behaviors predicted by the first‐order kinetic model

The kinetic data of the single‐factor experiment and orthogonal experiment on different parts of rosemary and the kinetic curves fitted by the first‐order kinetic model are shown in Figures 2 and 3. The extraction yield of essential oil from different parts of rosemary has an excellent linear relationship with DT (R^2^ > 0.98), which accords with the first‐order kinetic model. It can be observed from Figure 2 that the yield of REO gradually increases with the extension of distillation time. Under the same distillation time, the REO yield of fresh branches and existing shoots showed apparent advantages, which indicated that different parts of rosemary had a particular influence on the extraction yield of volatile oil and verified the conclusion of 3.2. With the extension of distillation time, the extraction content of fresh leaves was slightly higher than that of annual branches. According to the results of content determination, the final selection conditions were as follows: fresh rosemary leaves, distillation for 2 h, applied to the orthogonal test.

According to the optimization results of the orthogonal test, the distillation time (DT = 2 h) is taken as the abscissa, and the increase of REO yield is taken as the ordinate. The curve is drawn by nonlinear fitting (Figure 3). The kinetic model equation of REO yield is as follows:

(5) $$Y_{t}=0.0267\left(1‐e^{‐0.0074\mathrm{DT}}\right)$$

Y_t_ is the REO yield (%) of the device under the processing conditions, and DT is the distillation time. We can get the best treatment result through the orthogonal test, get a more considerable predicted value of rosemary essential oil yield after balance, and establish a characteristic kinetic model to describe the process better. Under this model, the rate of return after REO equilibrium can reach 2.67% theoretically. The results show that the regression coefficient *R* ^2^ is more significant than .95, which indicates that the equation is reliable, and the obtained results can be used for optimization. Under the support of this theory, the follow‐up research group used rosemary raw materials from the same origin to carry out the same experimental treatment, which was repeated three times. The final actual yield was between 2.52% and 2.73%, which was equivalent to the theoretical value, proved the treatment's reliability (Table 5).

**TABLE 5:** The first‐order kinetic model of experiment 2.3, DT is the distillation time, and the number is the fitting parameter

Factors	Fitting equation	R^2^
Fresh leaves	Y = 0.00008DT−0.0002	.9995
One‐year branches	Y = 0.00007DT+0.00005	.9937
perennial branches	Y = 0.00003DT−0.0003	.9883

## 4 CONCLUSION

Extraction of rosemary essential oil was studied in single‐factor experiments and orthogonal experiments and used the first‐order kinetics model to verify the data. The single‐factor experiments used different concentrations of NaCl and different parts of the rosemary plant to examine extraction conditions. Orthogonal tests examined water‐to‐material ratios, concentrations of NaCl, and integrity of leafy branches of rosemary. When extracting essential oil by hydrodistillation, the results show that it is preferred to add NaCl concentration at 5%, crushed the branches and leaves from the current year about 2 cm, and use a water‐to‐material ratio of 1:3 to obtain the high extraction yield. This is the result of economic and environmental considerations. In addition, according to the kinetic model, the yield under the optimum extraction conditions is estimated to be 2.67%. However, essential oil content in rosemary may be affected by many factors, such as plant growth conditions (including soil, climate, precipitation, etc.), so this study cannot wholly solve essential oil extraction. In conclusion, rosemary is a volatile oil product used widely in many fields and has significant economic value in the market. Our experiment revealed the best conditions for extracting rosemary essential oil by hydrodistillation.

## CONFLICT OF INTEREST

It is declared that there is no conflict of interest in the publication of this work.

## AUTHOR CONTRIBUTION

Hanyue Chen: Data curation (lead); Formal analysis (lead); Methodology (equal); Visualization (lead); Writing‐original draft (lead); Writing‐review & editing (equal). Zhanying Gu: Conceptualization (lead); Funding acquisition (lead); Investigation (equal); Methodology (equal); Project administration (lead); Resources (lead); Supervision (lead); Validation (equal); Writing‐review & editing (equal). Ling Yang: Investigation (equal); Methodology (equal). Ruonan Yang: Investigation (equal). Yaxin Ji: Investigation (equal). Qingyang Zeng: Investigation (equal). Peng Huang: Investigation (equal). Fangmeng Xiao: Investigation (equal).

## ETHICAL APPROVAL

Our research did not contain any animal experiments and human subjects.

## Data Availability

The data that support the findings of this study are available on request from the corresponding author. The data are not publicly available due to privacy or ethical restrictions.
